# Supplementary figures and images for: Bacterial Community Shifts during Polyp Bail-Out Induction in Pocillopora Corals
Source: Microbiol Spectr. 2023 Jun 28;11(4):e00257-23. doi: 10.1128/spectrum.00257-23 (PMC10433994; doi:10.1128/spectrum.00257-23)

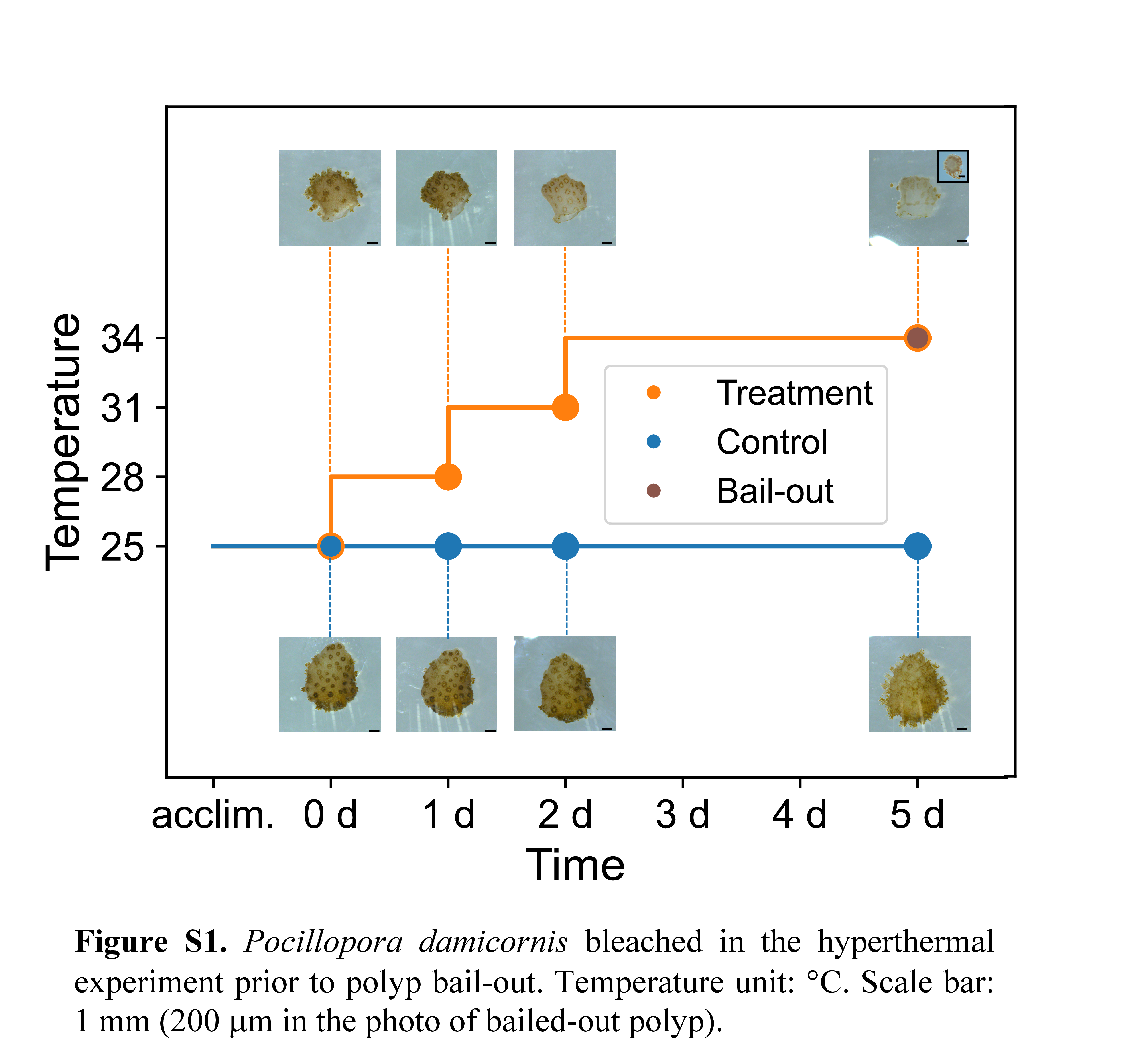

Supplement: Supplemental file 1 — Supplemental material. Download spectrum.00257-23-s0001.tif, TIF file, 4.2 MB [file spectrum.00257-23-s0001.tif]

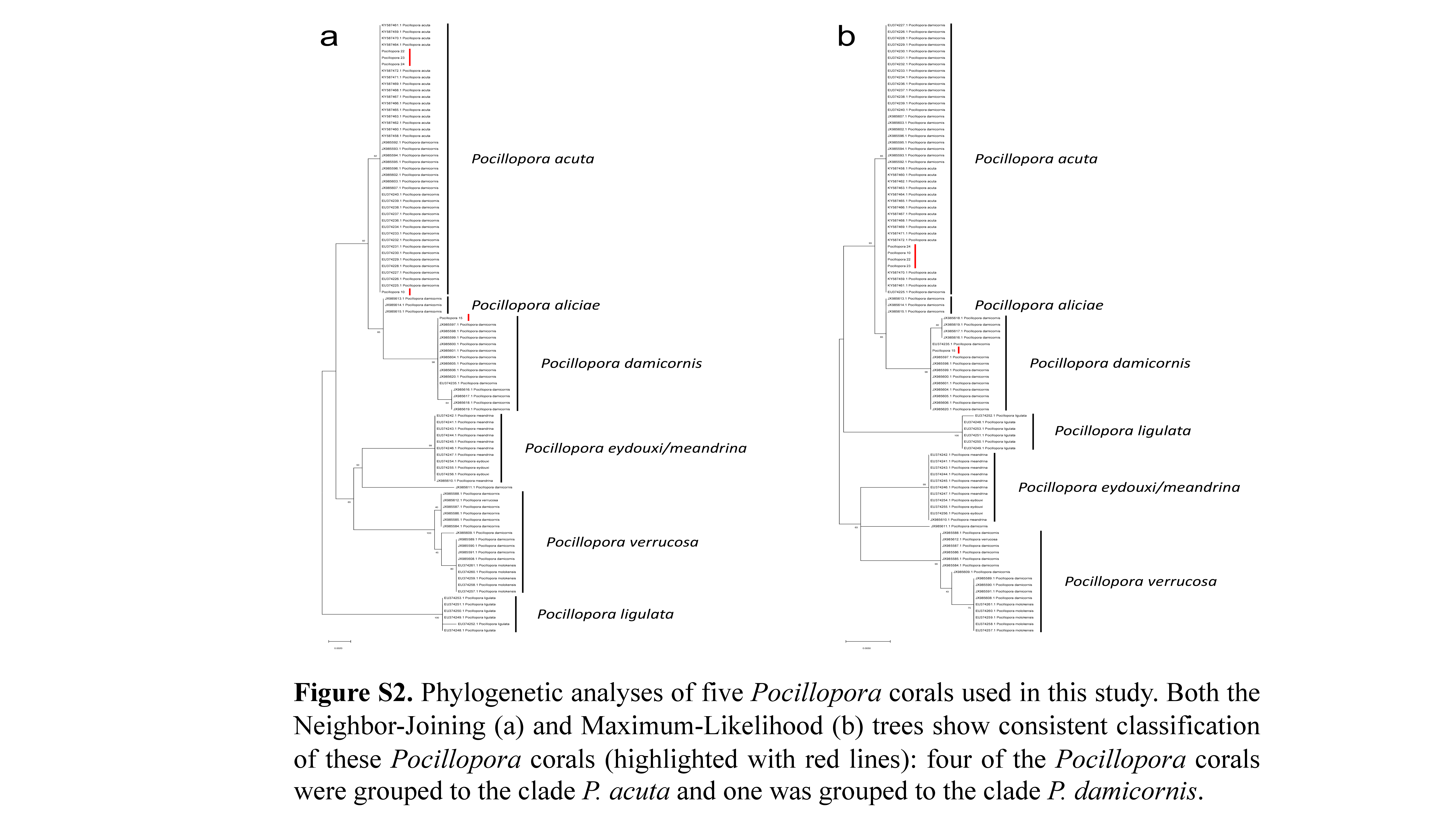

Supplement: Supplemental file 2 — Supplemental material. Download spectrum.00257-23-s0002.tif, TIF file, 1.5 MB [file spectrum.00257-23-s0002.tif]

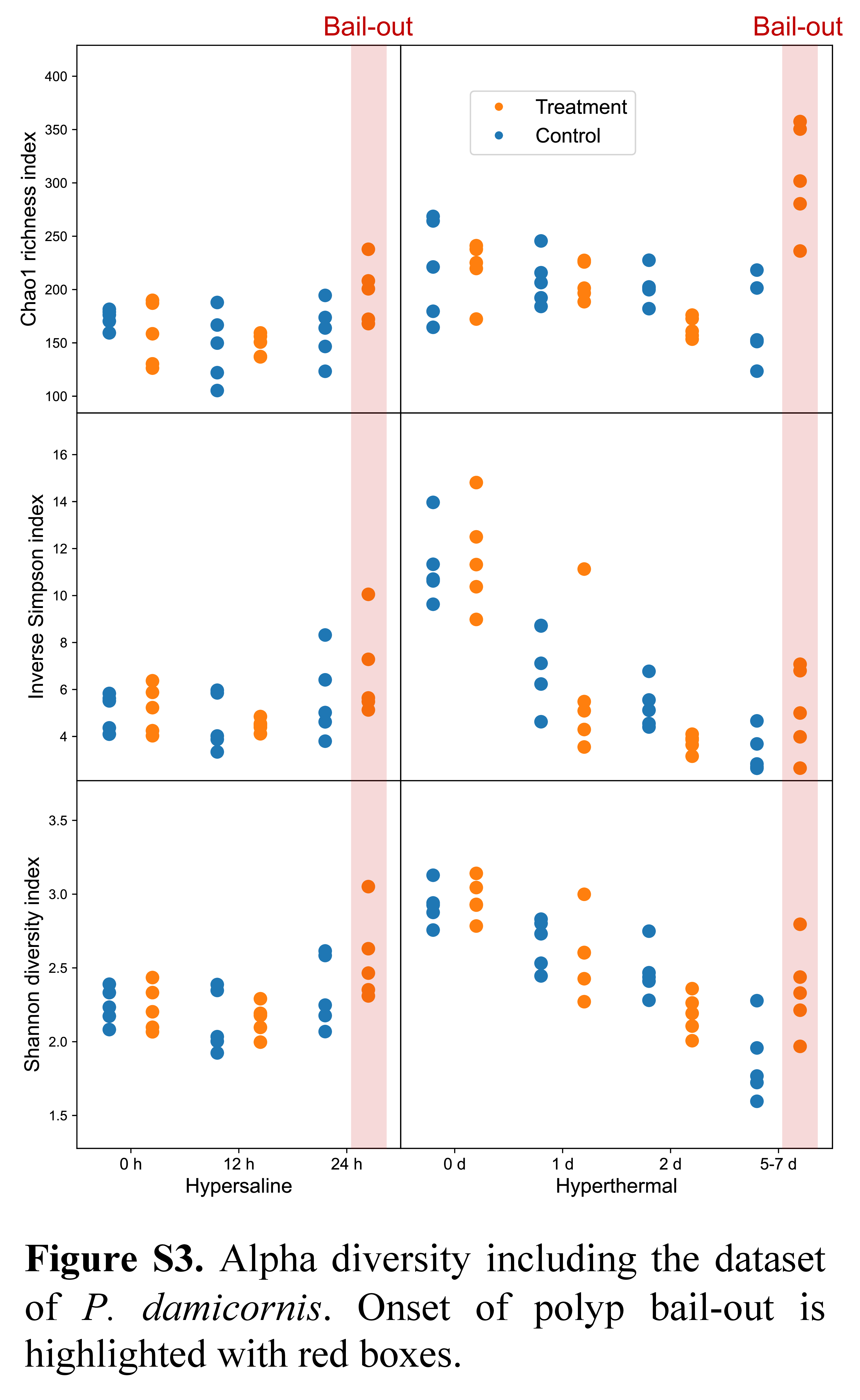

Supplement: Supplemental file 3 — Supplemental material. Download spectrum.00257-23-s0003.tif, TIF file, 0.6 MB [file spectrum.00257-23-s0003.tif]

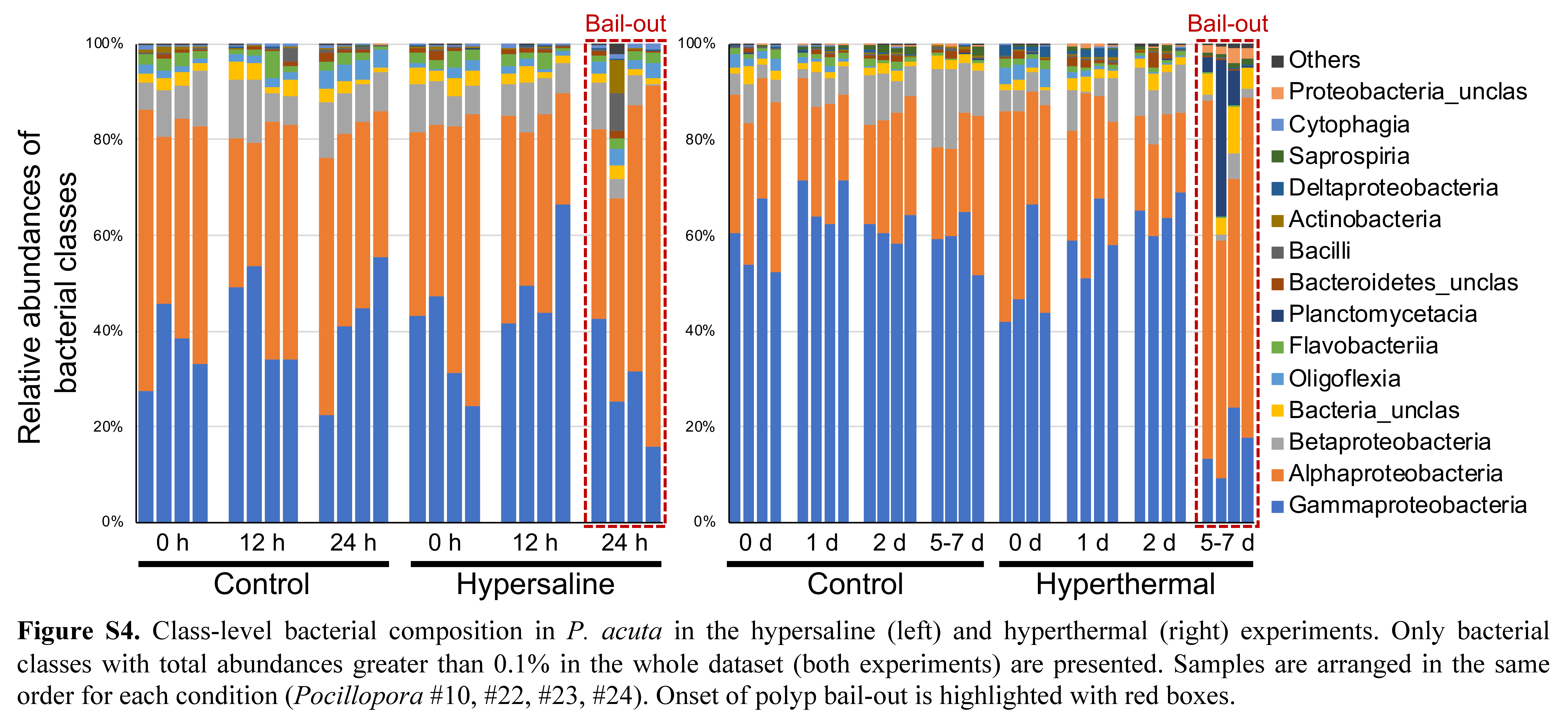

Supplement: Supplemental file 4 — Supplemental material. Download spectrum.00257-23-s0004.tif, TIF file, 2.1 MB [file spectrum.00257-23-s0004.tif]

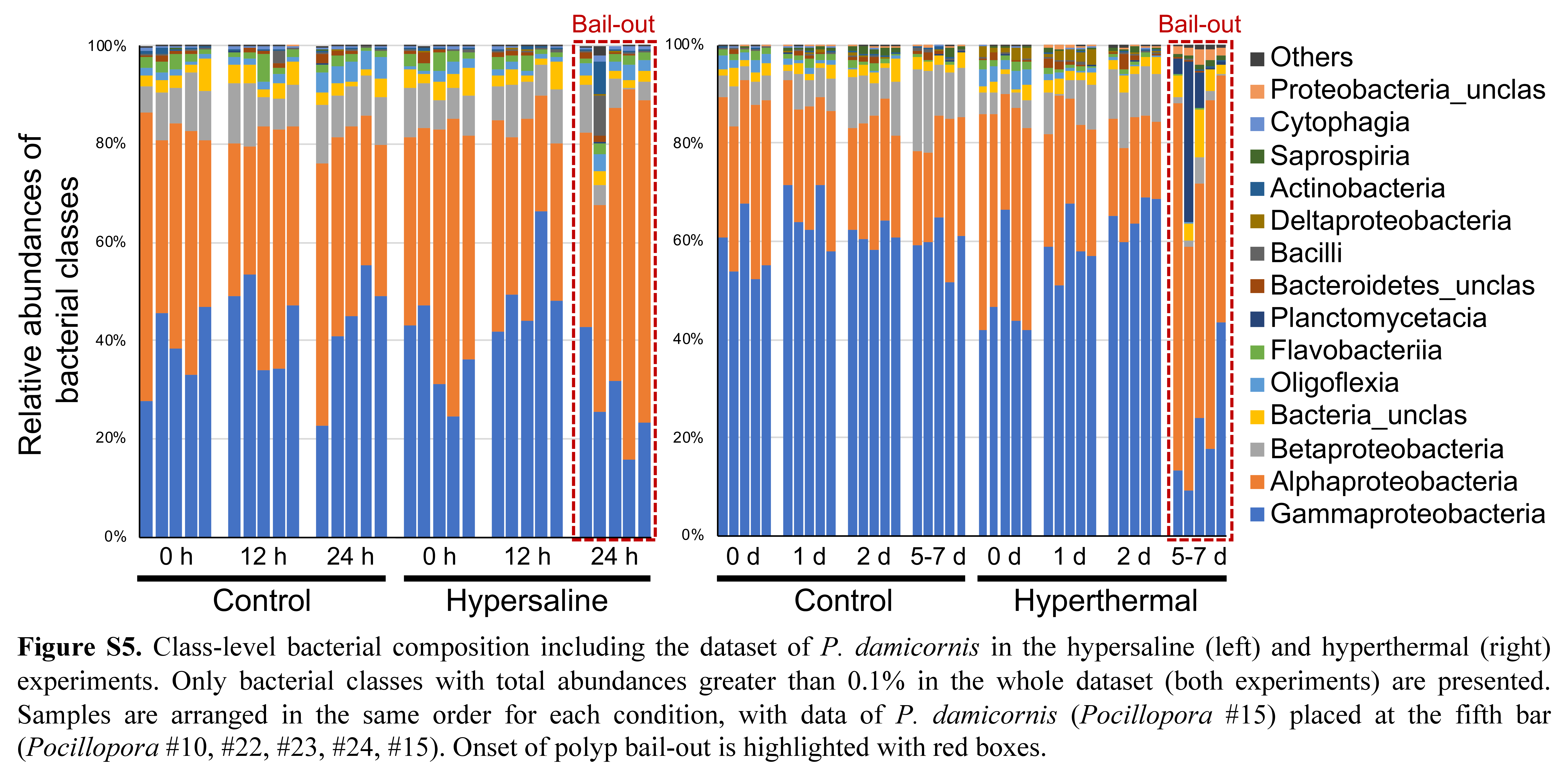

Supplement: Supplemental file 5 — Supplemental material. Download spectrum.00257-23-s0005.tif, TIF file, 2.5 MB [file spectrum.00257-23-s0005.tif]

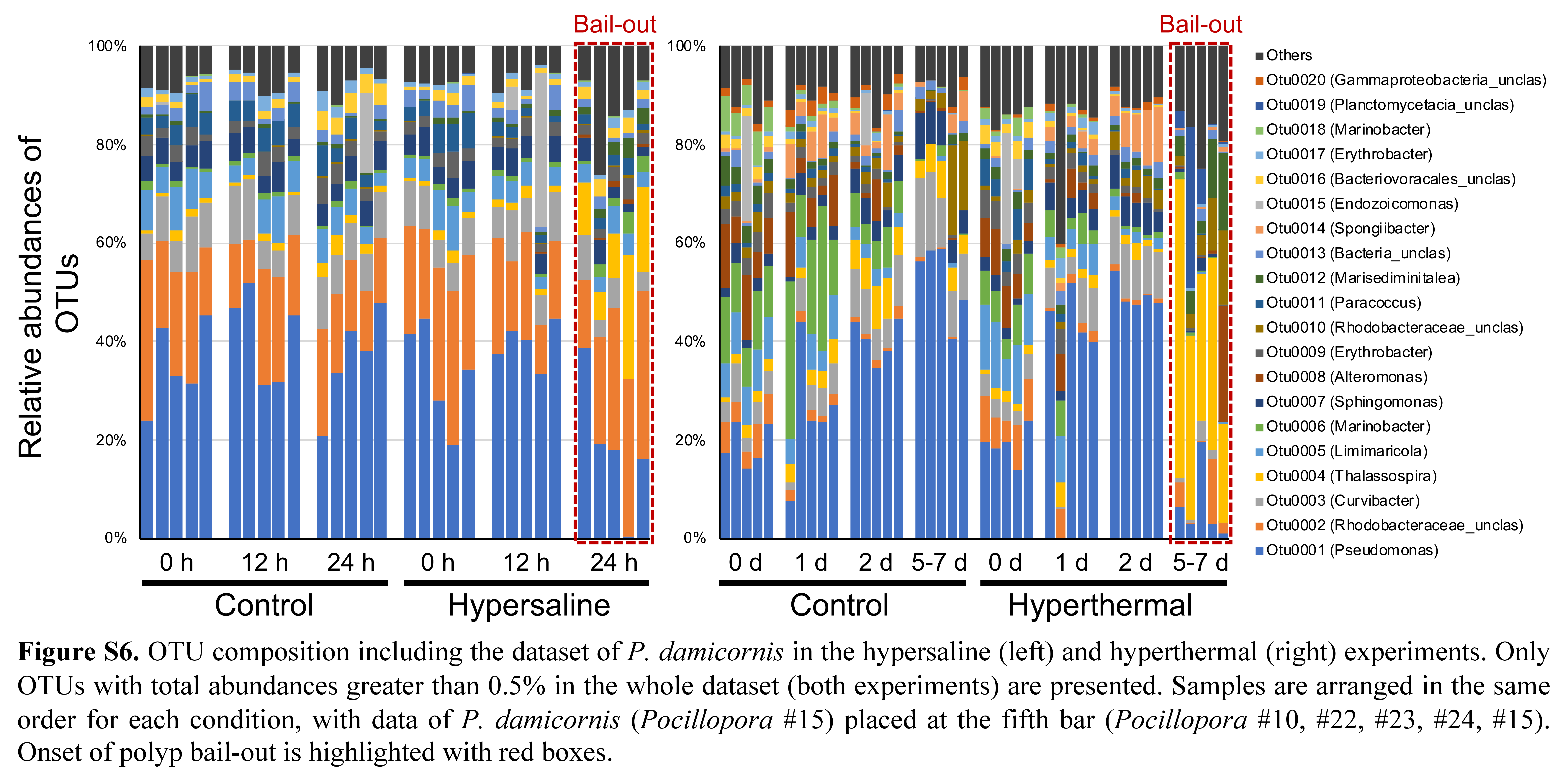

Supplement: Supplemental file 6 — Supplemental material. Download spectrum.00257-23-s0006.tif, TIF file, 2.7 MB [file spectrum.00257-23-s0006.tif]

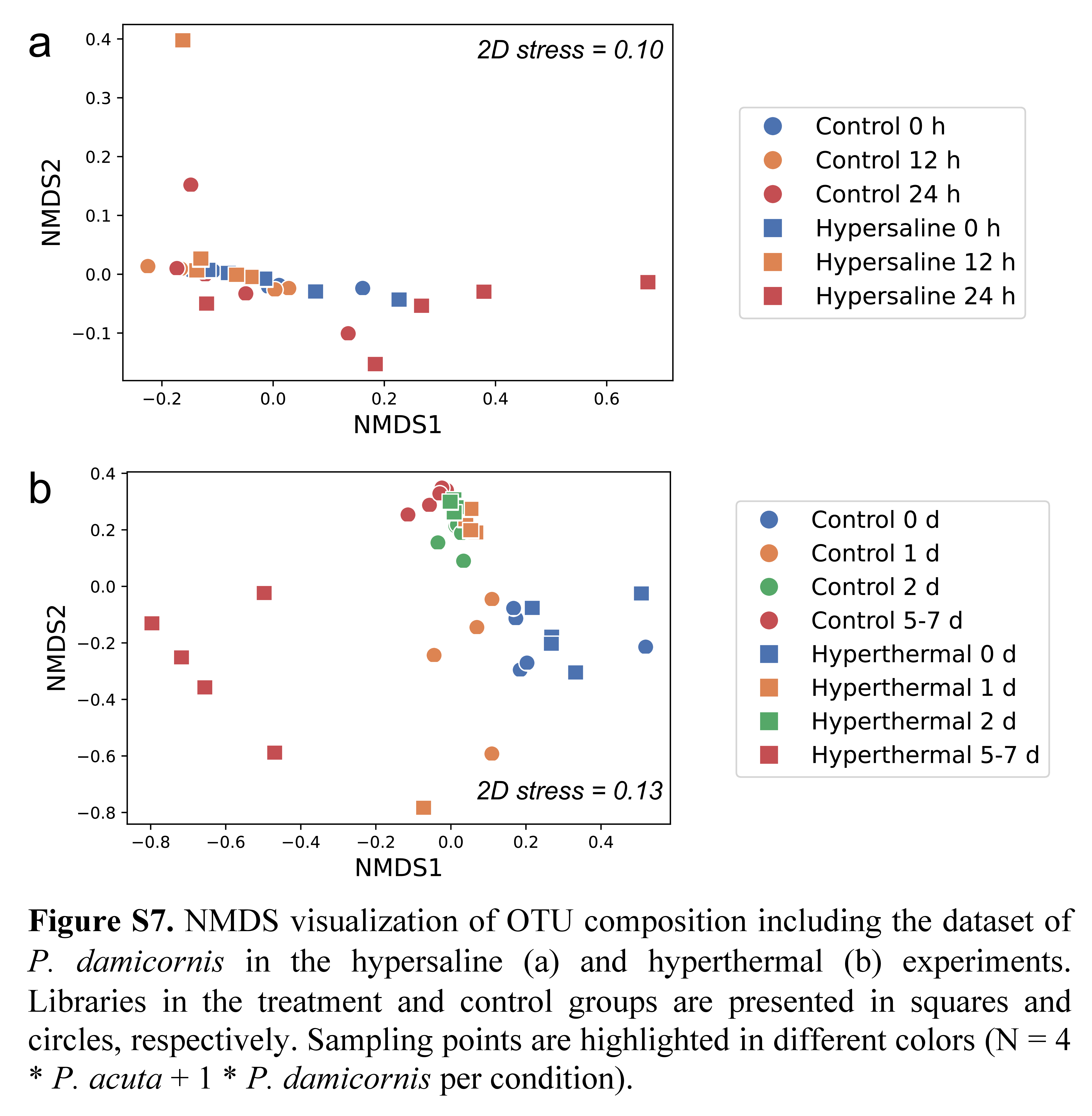

Supplement: Supplemental file 7 — Supplemental material. Download spectrum.00257-23-s0007.tif, TIF file, 0.9 MB [file spectrum.00257-23-s0007.tif]

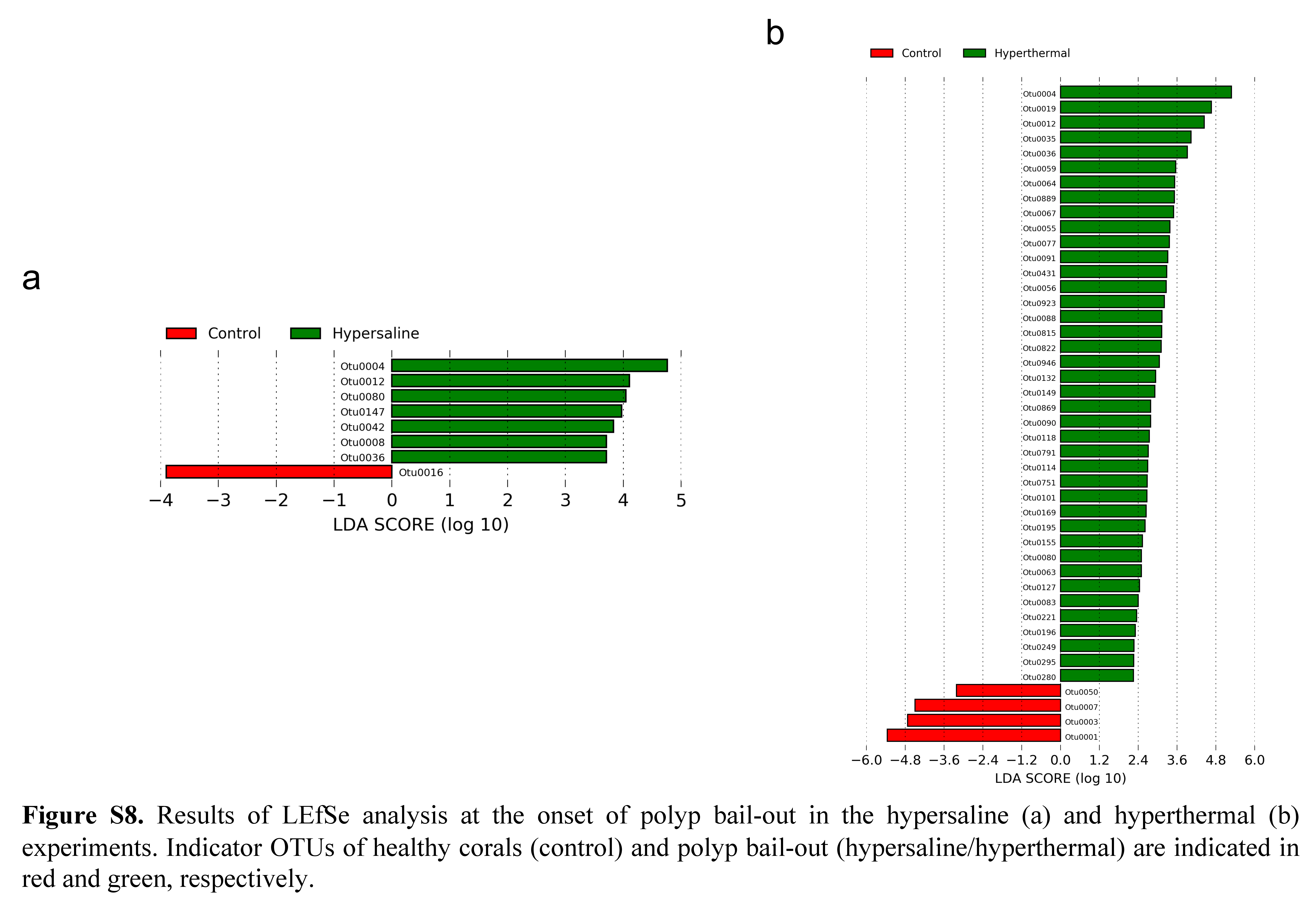

Supplement: Supplemental file 8 — Supplemental material. Download spectrum.00257-23-s0008.tif, TIF file, 1.9 MB [file spectrum.00257-23-s0008.tif]
